# Photobiomodulation by Led Does Not Alter Muscle Recovery Indicators and Presents Similar Outcomes to Cold-Water Immersion and Active Recovery

**DOI:** 10.3389/fphys.2018.01948

**Published:** 2019-01-14

**Authors:** Elvis de Souza Malta, Fabio Santos de Lira, Fabiana Andrade Machado, Anderson Saranz Zago, Sandra Lia do Amaral, Alessandro Moura Zagatto

**Affiliations:** ^1^Laboratory of Physiology and Sport Performance, Department of Physical Education, School of Sciences, São Paulo State University, Bauru, Brazil; ^2^Department of Physical Education, School of Technology and Sciences, São Paulo State University, Presidente Prudente, Brazil; ^3^Group of Studies and Research in Exercise Physiology Applied to Humans, Department of Physical Education, State University of Maringá, Maringá, Brazil; ^4^Department of Physical Education, School of Sciences, São Paulo State University, Bauru, Brazil

**Keywords:** low-level light therapy, high-intensity interval training, inflammation, interleukin-10, tumor necrosis factor-alpha, creatine kinase, L-lactate dehydrogenase

## Abstract

**Purpose:** The aim of the present study was to investigate the effectiveness of photobiomodulation therapy (PBMT) on muscle recovery based on inflammation (interleukin-10 [IL-10]; tumor necrosis factor-α [TNFα]), muscle damage markers (creatine kinase [CK]; lactate dehydrogenase [LDH]), delay onset muscle soreness (DOMS), and countermovement jump performance (CMJ) after two sprint interval training (SIT) sessions compared with a placebo condition (part-I), as well as to compare the effectiveness of PBMT with active recovery (AR) and cold-water immersion (CWI) (part-II).

**Methods:** Part-I was conducted as a double-blind, randomized and placebo-controlled study and part-II as a parallel-group study. Thirty-six men participated in the studies (12 participants in part-I and 36 participants in part-II). Volunteers performed two SITs interspaced by 24-h (SIT_1_ and SIT_2_) to mimic the effect of accumulating 2 consecutive days of SIT. In part-I, only after SIT_2_, PBMT [Total energy: 600J (300J per leg in 5 spots); wavelength: 660–850 nm] or placebo interventions were performed, while in part-II PBMT (part-I data), AR (15-min; 50% of the maximal aerobic power), or CWI (10-min; 10°C) were carried out, also after SIT_2_. Blood samples were collected before (i.e., baseline), and 0.5, 1, 24, 48, and 72-h after SIT_2_, while CMJ and DOMS were measured before, 24, 48, and 72-h after SIT_2_.

**Results:** In part-I, there were no interactions between PBMT and placebo conditions for any blood markers (*P* ≥ 0.313), DOMS (*P* = 0.052), and CMJ (*P* = 0.295). However, an effect of time was found with increases in LDH, CK, and IL-10 (*P* ≤ 0.043) as well as a decrease in DOMS at 72-h compared with 24-h (*P* = 0.012). In part-II, there were no interactions between the PBMT, AR, and CWI groups for any markers at the same moments (*P* ≥ 0.189) and for the peak and integral values (*P* ≥ 0.193), for DOMS (*P* = 0.314) and CMJ (*P* = 0.264). However, an effect of time was found with an increase in CK and IL-10 (*P* = 0.003), while DOMS decreased at 48 and 72-h compared with 24-h (*P* = 0.001).

**Conclusion:** In summary, PBMT had no effect on inflammation, muscle damage, CMJ performance, or DOMS after two consecutive sprint interval training sessions compared to placebo, CWI, and AR strategies.

## Introduction

Sprint interval training (SIT) is a time-efficient method of providing cardiorespiratory and muscular adaptations with a lower training volume (Gibala et al., 2012), in addition to which, it has also recently been suggested as an additional tool in the treatment of disease (Gibala et al., 2012). However, execution of this mode of training seems to be associated with a higher possibility of damage and inflammatory processes in muscular tissue, evidenced by increases in systemic biochemical markers and cytokine concentrations (Antosiewicz et al., 2013; Harnish and Sabo, 2016). Considering that during training planning, interest in the monitoring/measurement of the recovery status is growing (i.e., measurement of responses of autonomic nervous system by heart rate variability, training impulse, or RPE-session) with a focus on choosing the ensuring training load/stress (Heidari et al., 2018), the use of recovery modalities after exercise, aiming to speed up the process of tissue repair, may be a valid strategy to associate with SIT sessions (Barnett, 2006).

Traditionally, active recovery (AR) and cold-water immersion (CWI) have been widely used to accelerate muscular recovery after intense exercise sessions (Barnett, 2006). However, despite their popularity, the beneficial effects of CWI and AR have recently been questioned (Barnett, 2006; Roberts et al., 2015). In this way, photobiomodulation therapy (PBMT), a type of light therapy that utilizes non-ionizing and non-thermal light sources in the visible and infrared spectrum, eliciting photophysical and photochemical events on biological tissue (Anders et al., 2015), has attracted attention in the area of sports and health sciences. Some isolated studies have suggested its effects in reducing muscle damage markers (Baroni et al., 2010; De Marchi et al., 2012; de Paiva et al., 2016), attenuating or anticipating inflammatory responses (Amadio et al., 2015; Zagatto et al., 2016), and reducing some symptoms of inflammation such as delayed onset muscle soreness (DOMS) and loss of muscle function (Borges et al., 2013). However, despite these findings, the effects of PBMT on overall human muscle recovery (i.e., considering perceptive, physiological, and functional aspects) are contradictory and the actual effectiveness remains uncertain.

The majority of studies with PBMT have investigated its effects using isolated contractions and exercise-induced muscle damage protocols (Baroni et al., 2010; Borges et al., 2013; de Paiva et al., 2016), and only a few after a common high-intensity exercise session such as SIT. Additionally, some studies have compared the effects of PBMT with cryotherapy methods (de Paiva et al., 2016; De Marchi et al., 2017), however, without precise temperature control, a determinant parameter for its effectiveness (Machado et al., 2016), and no studies have compared PBMT with an AR protocol, a widely used method after exercise sessions. The possible beneficial effects of PBMT on overall muscle recovery may contribute to fortifying this method as an additional tool in the exercise routine.

Therefore, the aim of the present study was to investigate the effectiveness of PBMT on muscle recovery in view of systemic inflammation (interleukin-10 [IL-10] and tumor necrosis factor-α [TNFα]), muscle damage (creatine kinase [CK] and lactate dehydrogenase [LDH]), DOMS, and muscle performance (countermovement jump performance [CMJ]) after SIT, and to compare PBMT with AR and CWI interventions. The hypothesis of the study was that PBMT would decrease CK and LDH blood concentrations, accelerate systemic inflammatory responses, and reduce DOMS and loss in CMJ performance.

## Materials and Methods

The study was conducted in two parts. Part-I was performed to compare the PBMT with the placebo (PLA) condition in a double-blind design, while part-II aimed to compare PBMT with AR and CWI with parallel groups.

### Participants

The minimum sample size for a statistical power of 90 % (alpha: 0.05; allocation ratio: 1) was 10 participants in each group. The sample size was calculated based on the findings of De Oliveira et al. (2018), using the TNFα results and assuming an effect size of 1.4 (*d* value). Thus, a total of thirty-six healthy men participated in the present investigation, of which twelve participated in both part-I and part-II, with an addition of twenty-four volunteers in part-II (i.e., total of thirty-six in part-II, allocated into three groups of twelve participants each).

Prior to beginning the study, volunteers were informed about the procedures, risks, and benefits involved in the tests and then signed the consent form. All experimental procedures were approved by the Human Research Ethics Committee from the School of Sciences, São Paulo State University – UNESP (protocol number: 1.139.070) and conducted in accordance with the Declaration of Helsinki.

Participants were untrained, healthy individuals, without any vascular disease, metabolic disorders, recent muscle-skeletal or joint injuries (i.e., in the previous 6 months), and had not used nutritional or pharmacological substances for at least 3 months. Volunteers who were regularly absent from the trials, initiated the use of nutritional and/or pharmacological substances during the evaluations, or presented muscle injury were excluded from the study.

### Experimental Design

Participants arrived at the laboratory in the morning after fasting (≈8-h). One hour before the evaluations an individual breakfast was offered to the volunteers composed of 30% of daily caloric expenditure (Mifflin et al., 1990).

The graded exercise test (GXT) and SIT were performed on an electromagnetic cycle ergometer (Excalibur Sport, Lode, Netherlands). Before all tests, a 5-min warm-up at 30% of the maximal aerobic power (MAP) reached in the GXT was performed.

The study was divided into two sequenced and dependent parts (I and II).

Part-I was conducted in a double-blind, randomized, and placebo-controlled design. Firstly, a GXT was performed to determine the peak oxygen uptake (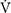
O_2peak_) and the MAP. Five days after the GXT, two SIT sessions (SIT_1_ and SIT_2_) were performed, interspaced by 24-h of recovery (double SIT), to potentiate the stress on active muscle. The double SIT (i.e., set of 2 SIT sessions) was performed twice before each experimental condition (i.e., PBMT and placebo), separated by 5-days. Immediately after the double SIT (i.e., only post the SIT_2_), PBMT was applied in mode on (PBMT condition – C_PBMT_) or off (PLA condition – C_PLA_) in randomized and counterbalanced order. To ensure blinding in each experimental condition, participants were blindfolded and wore headphones to eliminate light and sound signals. A person not involved in any parts of the study applied the PBMT and randomization.

Part-II was conducted as a parallel-group trial, with GXT and SIT sessions identical to those reported in part-I; however, immediately after the double SIT (i.e., only post SIT_2_), the recovery interventions were composed of active recovery (AR; group AR – G_AR_) or cold-water immersion (CWI; group CWI- G_CWI_). Posteriorly, the C_PBMT_ data recorded from part-I were used to compare with G_AR_ and G_CWI_ in part-II.

Venous blood sample collections were realized in the medial cubital vein at rest (i.e., baseline) and 0.5, 1, 24, 48, and 72-h after each recovery condition (part-I) or intervention (part-II) using vacutainer tubes of 10 and 4 mL (BD, Juiz de Fora, MG, Brazil) for inflammatory and muscle damage marker measurements. Capillary blood samples (25 μL) were collected from the earlobe 3, 5, and 7-min after GXT, and before, between intervals (i.e., 3rd-min after each Wingate test) and 5 and 7-min after each SIT session for measurement of lactate concentrations.

In parts I and II, CMJ performance and DOMS were evaluated at rest (i.e., baseline), and 24, 48, and 72-h after interventions.

**FIGURE 1:**
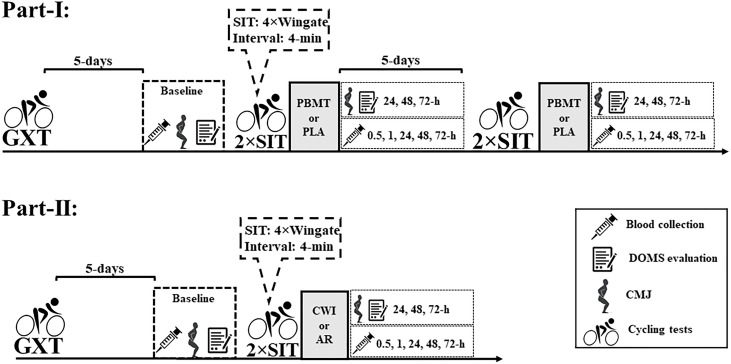
Experimental design of studies I and II. DOMS, delay onset muscle soreness; CMJ, countermovement jump; PBMT, photobiomodulation therapy; CWI, cold-water immersion; AR, active recovery; PLA, placebo; GXT, graded exercise test; 2 × SIT, double sprint interval training.

Figure 1 presents the experimental design of studies-I and II.

### Graded Exercise Test

Graded exercise test was performed to determine the 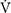
O_2peak_ and MAP, starting at 75 W, with a 25 W increment every 2-min until exhaustion (Howley et al., 1995; Ozyener et al., 2001), measured at 670-m above sea level. During GXT, respiratory responses were registered breath-by-breath using a gas analyzer (Quark CPET, COSMED, Italy), previously calibrated in accordance with the manufacturer’s instructions. Data were smoothed every 10-points and interpolated every 1-s using the software OriginPro 8.0 (OriginLab Corporation, United States). For 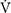
O_2peak_ determination, the oxygen uptake (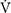
O_2_) mean of the final 20-s of each stage was determined and the 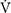
O_2peak_ was assumed as the highest 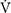
O_2_ mean reached in the GXT. MAP was recorded and considered the highest exercise power performed during the test.

### Sprint Interval Training Session

The double SIT comprised two SIT sessions interspaced by 24-h, mimicking the accumulated effects of two consecutive training days. Each SIT constituted four Wingate tests (i.e., 30-s at 0.7 Nm⋅kg^-1^) with a 4-min recovery between bouts (Burgomaster et al., 2005). In the first minute of recovery time an active recovery at 30% of MAP and ≈75–80 rpm was performed to minimized is comfort, while the additional recovery time (i.e., 3-min) was composed of passive recovery (Gibala et al., 2012). The SIT protocol was controlled using Wingate 1.11 software (Lode, Netherlands) which enabled measurement of bout work (BW), peak power (PP), and mean power (MP). Workload performed during the double SIT was assumed as the sum of all work performed during the Wingate tests (total work – TT). In a previous study performed in our laboratory, the SIT showed good reliability of TT 7-days after a first session (ICC = 0.89) (Malta et al., 2018). The SIT performance parameters were measured to ensure that the volunteers were submitted to the same exercise effort in both study parts.

### CMJ and DOMS Measurements

To evaluate the symptoms of inflammation (i.e., muscle functional limitation and DOMS), the CMJ and DOMS were measured. CMJ was composed of 3 maximal jump trials interspaced by 1-min of passive recovery. Volunteers were instructed to remain with hands on hips and flex the knees quickly to 90° to jump. To assess the jump height, a jump platform was used (Jump test, Cefise, Brazil) and the highest jump was considered. This configuration of CMJ test was chosen as it does not influence blood cytokines or muscle damage markers. In addition, DOMS perception was assessed using a VAS consisting of a 100 mm line, on which the “0” represents “no pain” and “100” “very painful” (Carlsson, 1983). For greater leg pain perception, the scale was applied during low-intensity pedaling (Borges et al., 2013). In the present study, CMJ and DOMS values are presented as change related to baseline (Δ), being described using the variable acronym plus the “Δ” (i.e., CMJ^Δ^ and DOMS^Δ^).

### Recovery Methods

**FIGURE 2:**
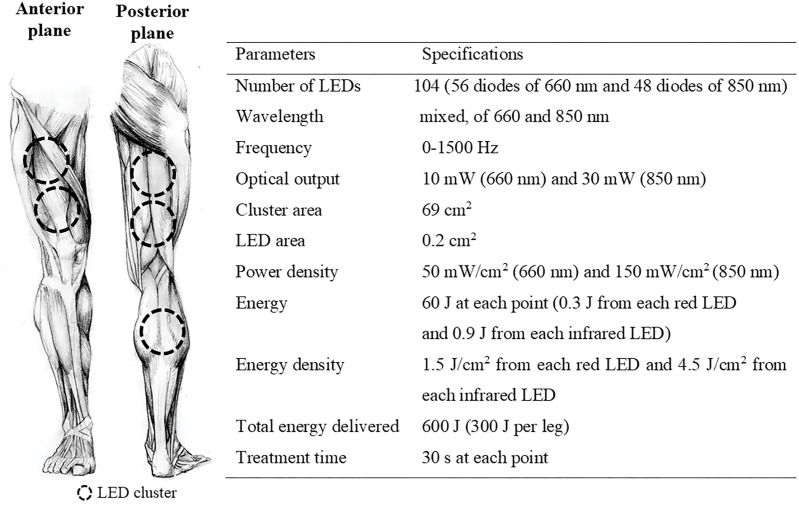
Technical parameters of PBMT and location of LED irradiation. LED, light emitting diode.

Photobiomodulation therapy was applied using a cluster multi-diode containing 104 LED (THOR-LX2, THOR Photomedicine Ltd., United Kingdom). The PBMT protocol had an overall duration of 2.5-min, with application in both legs simultaneously. LED irradiation was performed in two regions of the quadriceps muscle, two regions of the biceps femoris, and one region between the soleus and gastrocnemius muscles, following the distribution axis of the muscle fibers. The interventions were performed using the spot method, with direct contact (i.e., 90° angle) of the equipment on the skin surface. The technical parameters of PBMT and location of LED irradiation are shown in Figure 2.

Active recovery and CWI were applied only in part-II. AR was performed on a cycle ergometer, immediately after the double SIT, with a duration of 15-min and intensity corresponding to 50% of MAP (Wigernaes et al., 2001). During AR, a cadence of ≈75 rpm was maintained. The partial CWI, to the waist, was performed immediately after the double SIT with the volunteers sitting in an immersion bath containing 200 L of water, at 10°C for 10-min (Machado et al., 2016). The temperature was set using an auto-cooling system, controlled by a digital thermostat (TIC17RGTi, ES Full Gauge, United States), triggered when the water temperature increased 0.1°C.

### Blood Sample Analysis

Capillary blood samples were deposited into microtubes containing 50 μL of sodium fluoride at 1% and analyzed in an electrochemical analyzer (YSI 2300 Stat Plus, Yellow Springs Instruments, United States) for determination of lactate concentrations. According to the manufacturer, the equipment has a measurement error of ±2%.

Venous blood samples were centrifuged for 10-min at 1306 *g* and 4°C (Vision scientific, VS-15000FNII, SKR) for extraction of plasma and serum. Plasmatic cytokines (IL-10 and TNFα) were assessed using ELISA commercial kits (Affymetrix, eBioscience, United States; Lot: IL-10 4295480; TNFα 4298657). Plates were read using a spectrophotometer, SpectraMax Plus 384 (Molecular Devices, United States). Serum CK and LDH concentrations were assessed through a kinetic method using a random-access analyzer (A-15, Biosystems S.A., Spain) and commercial kits (Biosystems S.A., Spain; Lot: CK 13869; LDH 09998).

Considering the great individual variation in CK, LDH, IL-10, and TNFα blood concentrations, in the present study the blood markers are presented as percentage difference to baseline (Δ%), being described using the variable abbreviation plus the “Δ%” (i.e., CK^Δ%^, LDH^Δ%^, IL-10^Δ%^, and TNFα^Δ%^). The integrals of CK, LDH, IL-10, and TNFα (i.e., area under the curve considering its concentration and the evaluation time) were calculated using the software OriginPro 8.0 (OriginLab Corporation, United States) to test whether exposure to inflammation and muscle damage were affected by recovery methods (Krzanowski and Hand, 2009). In addition, peak concentration of CK, LDH, IL-10, and TNFα (i.e., highest value obtained between 0.5 and 72-h) were calculated to measure the magnitude of increase.

### Statistical Analysis

Statistical analyses were performed using the software package SPSS version 23.0 (IBM Corp., Armonk, NY, United States). Data are presented as means and standard deviations (mean ± SD). The normality of the data was confirmed using the Shapiro-Wilk test. In both parts (e.g., I and II), to compare the CK^Δ%^, LDH^Δ%^, IL-10^Δ%^, and TNFα^Δ%^ blood concentrations, DOMS^Δ^ and CMJ^Δ^ performance between moments (i.e., main time effect) and between and within-conditions and groups (i.e., interaction time^∗^groups) a two-way repeated measure ANOVA was used and a SIDAK *post hoc* was applied when necessary. Mauchly’s sphericity test was used in all ANOVA analyzes, and in cases of sphericity violation, the F and significance corrected by Greenhouse-Geisser were assumed. Only in part-I, the paired *t*-test was used to compare the means of SIT total work, integral, and peak blood concentration of CK^Δ%^, LDH^Δ%^, IL-10^Δ%^, and TNFα^Δ%^ (i.e., C_PBMT_ × C_PLA_). In addition, the *t*-test was also used to compare peak lactate concentrations which were measured two times for each condition (i.e., C_PBMT_ SIT_1_ × SIT_1_ C_PLA_ and C_PBMT_ SIT_2_ × SIT_2_ C_PLA_). In part-II, the ANOVA one-way test was used to compare peak lactate concentrations, MAP, 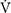
O_2peak_, and integral and peak blood markers between groups (i.e., C_PBMT_, G_AR_, and G_CWI_). In both parts (e.g., I and II), SIT total work and peak lactate concentration were compared to verify similar exercise-induced workload and metabolic stress. In all cases, a significance level of 5% was assumed.

## Results

### Part-I and II: GXT and SIT Outcomes

In part-I, the 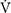
O_2peak_ and MAP reached in the GXT were 40.0 ± 5.7 mL⋅kg^-1^⋅min^-1^ and 210.4 ± 29.1 W, respectively. The double SIT total work (i.e., SIT_1_ and SIT_2_ work sum) for C_PBMT_ and C_PLA_ were 96.4 ± 13.4 and 99.5 ± 13.9 kJ, respectively. In addition, the peak lactate concentrations reached in SIT for C_PBMT_ and C_PLA_ were 14.1 ± 2.7 and 14.4 ± 2.0 (SIT_1_), 13.9 ± 2.9, and 14.2 ± 2.0 mmol⋅L^-1^ (SIT_2_), respectively. No differences between C_PLA_ and C_PBMT_ were verified in double SIT total work [*P* = 0.941, *t*_(11)_ = -0.075] or peak lactate concentration reached after SIT sessions [SIT_1_: *P* = 0.479, *t*_(9)_ = -0.738; SIT_2_: *P* = 0.666; *t*_(11)_ = -0.444].

In part-II, there were no significant differences between the anthropometric characteristics of the volunteers [*P* ≥ 0.136; *F*_(2,33)_ ≤ 2.123] (Table 1). The 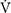
O_2peak_ reached in GXT for G_AR_ and G_CWI_ were 41.9 ± 5.0, 38.1 ± 6.5 mL⋅kg^-1^⋅min^-1^, while MAP values were 218.8 ± 45.4 and 214.6 ± 45.8 W, respectively. No significant differences were verified between groups (i.e., C_PBMT_, G_AR_, and G_CWI_) in 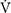
O_2peak_ [*P* = 0.306; *F*_(2,33)_ = 1.229] and MAP [*P* = 0.883; *F*_(2,33)_ = 0.125] reached in the GXT. The SIT total work for G_AR_ and G_CWI_ were 103.1 ± 25.8, and 99.6 ± 21.8 kJ. In addition, the peak lactate concentrations reached in SIT for G_AR_ and G_CWI_ were 14.7 ± 1.2 and 14.3 ± 0.9 mmol⋅L^-1^ (SIT_1_), and 14.0 ± 1.4 and 13.5 ± 1.4 mmol⋅L^-1^ (SIT_2_), respectively. No differences between C_PBMT_, G_AR_, and G_CWI_ were verified in double SIT total work [*P* = 0.874; *F*_(2,33)_ = 135] and peak lactate concentration reached after the SIT session [SIT_1_: *P* = 0.859; *F*_(2,31)_ = 0.152 and SIT_2_: *P* = 0.889; *F*_(2,33)_ = 0.118].

**Table 1:** Anthropometric characteristics of the volunteers.

	Part-I	Part-II		ANOVA one-way		
		G_CWI_	G_AR_	*P*-value	*F*	Df
Age (years)	25.7 ± 5.1	23.7 ± 4.4	24.2 ± 5.5	0.606	0.509	2, 33
Height (cm)	177.3 ± 3.0	175.5 ± 4.1	179.7 ± 7.0	0.229	1.541	2, 33
Weight (kg)	76.3 ± 7.4	73.9 ± 7.8	73.6 ± 10.0	0.597	0.524	2, 33
BMI (kg/m^2^)	24.2 ± 1.7	25.4 ± 4.2	22.8 ± 2.9	0.136	2.123	2, 33


*G_CWI_, group submitted to cold-water immersion; G_AR_, group submitted to active recovery; BMI, body mass index; Df, degrees of freedom.*

Additional performance parameters (i.e., BW, PP, and MP) and lactate kinetics before and during, and the peak reached after double SIT sessions for parts I and II are shown in Supplementary Figures S1, S2, respectively.

### Part-I: Recovery Outcomes

**FIGURE 3:**
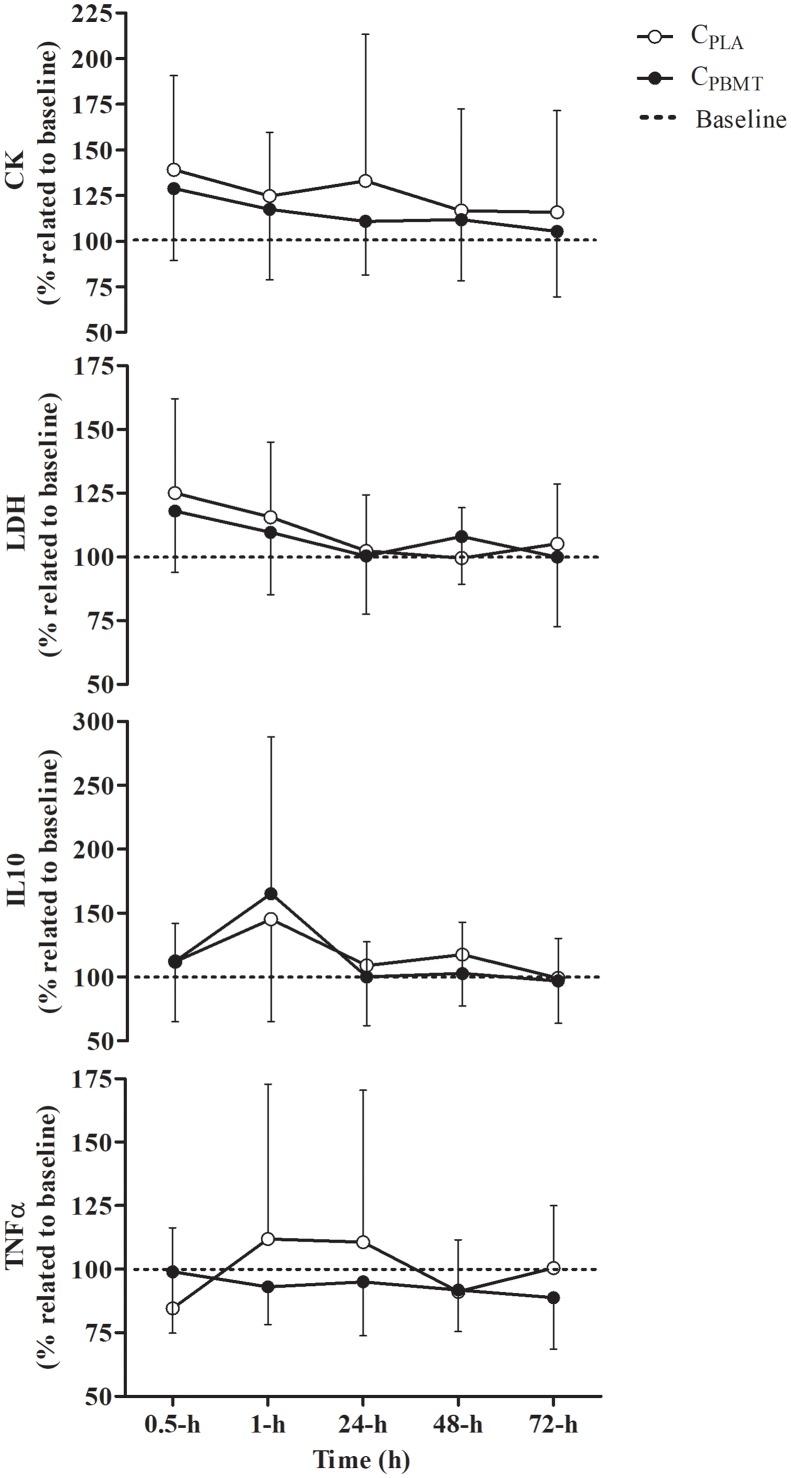
Blood concentration of IL-10^Δ%^, TNFα^Δ%^, CK^Δ%^, and LDH^Δ%^ (mean ± SD) for C_PBMT_ and C_PLA_. IL-10^Δ%^, interleukin 10 expressed as percentage difference to baseline; TNFα^Δ%^, tumor necrosis factor alpha expressed as percentage difference to baseline; CK^Δ%^, creatine kinase expressed as percentage difference to baseline; LDH^Δ%^, lactate dehydrogenase expressed as percentage difference to baseline; C_PBMT_, photobiomodulation therapy condition; C_PLA_, placebo condition.

There was a time effect showing kinetic changes in some blood markers, independent of the treatment, but no effect for TNFα^Δ%^ blood concentration [*P* = 0.668; *F*_(4,32)_ = 0.324]. The LDH^Δ%^ increased at 0.5-h compared to 24, 48, and 72-h [*P* = 0.000; *F*_(4,36)_ = 7.035; *post hoc* *P* ≤ 0.030]. The CK^Δ%^ decreased over time at 72-h compared with 0.5-h [*P* = 0.043; *F*_(4,40)_ = 2.716; *post hoc* *P* = 0.021]. The IL-10^Δ%^ also decreased over time at 24 and 72-h compared with 1-h [*P* = 0.035; *F*_(4,40)_ = 3.944; *post hoc* *P* ≤ 0.048]. Contrarily, there were no interactions (i.e., interaction time^∗^groups) between C_PBMT_ and C_PLA_ for any blood markers [*P* ≥ 0.313; *F*_(4,32)_ ≤ 1.327] (Figure 3). The part-I absolute values of CK, LDH, IL-10, and TNFα blood concentrations are presented in Supplementary Table S1.

Table 2 presents the peak and integral values of CK^Δ%^, LDH^Δ%^, IL-10^Δ%^, and TNFα^Δ%^ reached in C_PBMT_ and C_PLA_. For all blood markers, there were no significant differences between C_PBMT_ and C_PLA_ for peak [*P* ≥ 0.104; *t*_(11)_ = -1.774] or integral values [*P* ≥ 0.370; *t*_(8)_ = -0.950].

**Table 2:** Comparison between C_PBMT_ and C_PLA_ for peak and integral values of CK, LDH, IL-10, and TNFα.

	C_PLA_		C_PBMT_		*t*-test			
	Peak (Δ%)	Integral (Δ%⋅h)	Peak (Δ%)	Integral (Δ%⋅h)	Peak		Integral	
					*P*-value (t)	Df	*P*-value (t)	Df
CK	170.6 ± 81.0	8330.5 ± 3308.6	139.7 ± 40.7	7902.5 ± 2170.3	0.10 (–1.774)	10	0.47 (–0.756)	10
LDH	132.1 ± 32.6	7442.6 ± 1469.7	129.1 ± 23.2	7427.3 ± 1332.8	0.28 (–0.358)	10	0.93 (–0.091)	9
IL-10	172.9 ± 70.2	8314.5 ± 2648.1	184.5 ± 110.7	7956.7 ± 2642.4	0.75 (0.323)	10	0.72 (–0.370)	10
TNFα	127.1 ± 57.5	6687.8 ± 1169.0	111.3 ± 23.1	6498.7 ± 983.0	0.29 (–1.119)	9	0.37 (–0.950)	8


*IL-10, interleukin 10; TNFα, tumor necrosis factor alpha; CK, creatine kinase; LDH, lactate dehydrogenase; C_*PBMT*_, condition photobiomodulation therapy; C_*PLA*_, condition placebo; Δ%, percentage alteration related to baseline;Δ%⋅h, percentage alteration related to baseline per hour; Df, degrees of freedom.*

**FIGURE 4:**
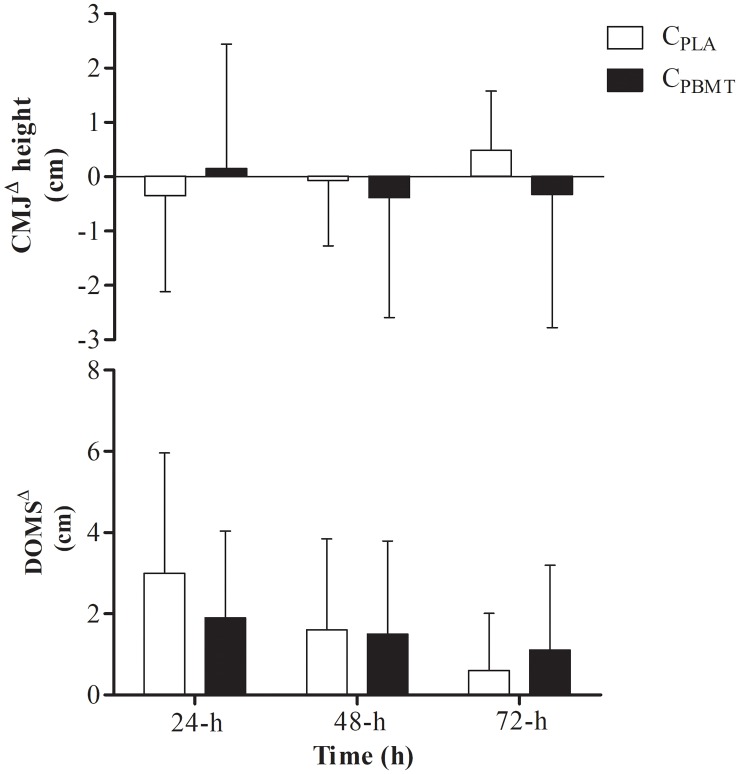
DOMS^Δ^ performance and DOMS^Δ^ for G_PBMT_ and G_PLA_ at 24, 48, and 72-h after double SIT session. DOMS^Δ^, delay onset muscle soreness expressed as difference to baseline; CMJ^Δ^, countermovement jump performance expressed as difference to baseline; C_PBMT_, condition photobiomodulation therapy; C_PLA_, condition placebo.

For DOMS^Δ^, a time effect was found for DOMS^Δ^ that decreased at 72-h compared with 24-h [*P* = 0.012; *F*_(2,22)_ = 7.263; *post hoc* *P* = 0.043], however, there were no interactions (i.e., interaction time^∗^groups) between C_PBMT_ and C_PLA_ at the same moments as for DOMS^Δ^ [*P* = 0.052; *F*_(2,22)_ = 4.298] and for CMJ^Δ^ [*P* = 0.295; *F*_(2,22)_ = 1.289] (Figure 4).

### Part-II: Recovery Outcomes

**FIGURE 5:**
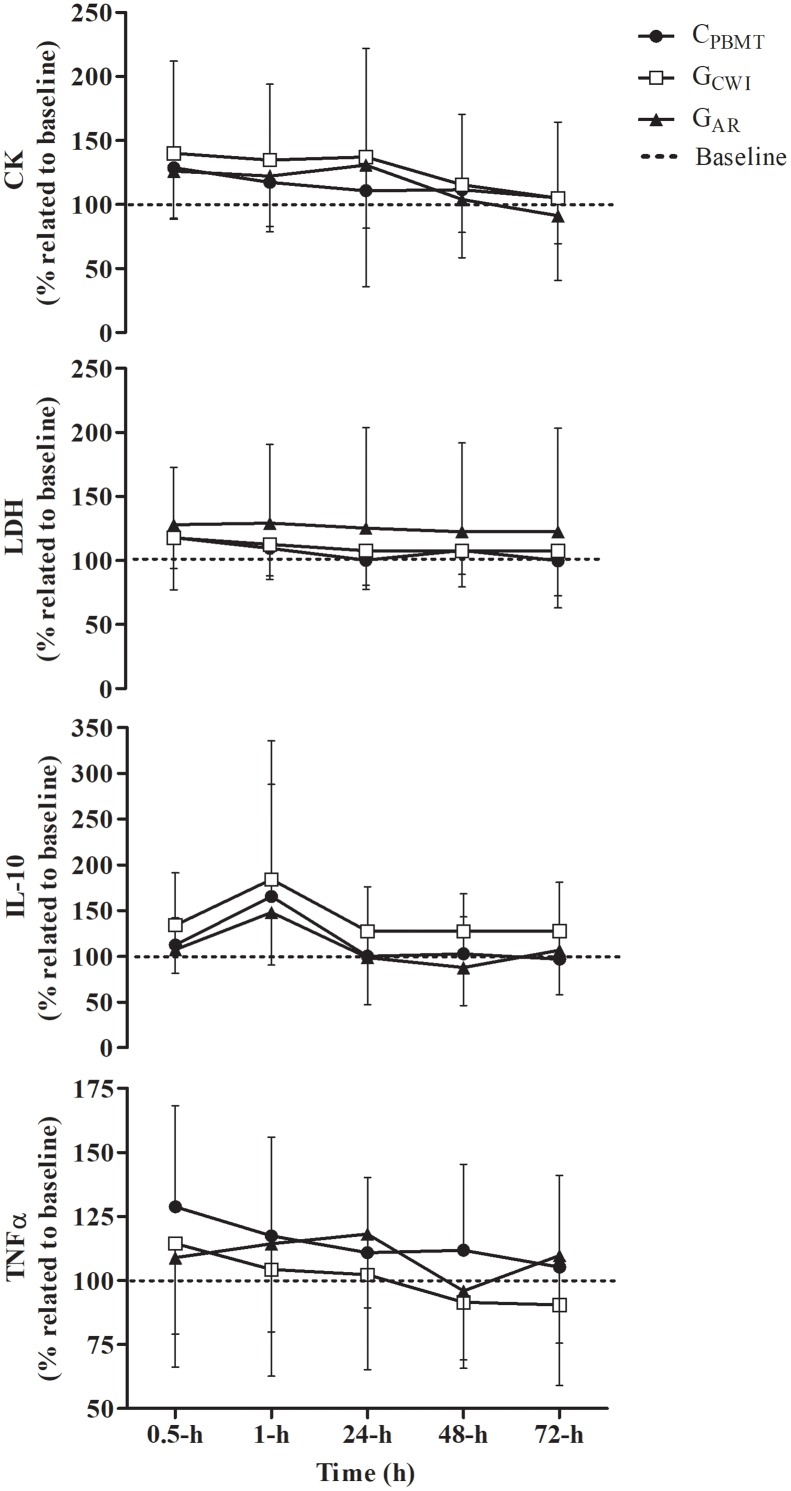
Blood concentration of IL-10^Δ%^, TNFα^Δ%^, CK^Δ%^, and LDH^Δ%^ (mean ± SD) for C_PBMT_, G_AR_, and G_CWI_. IL-10^Δ%^, interleukin 10 expressed as percentage difference to baseline; TNFα^Δ%^, tumor necrosis factor alpha presented as percentage difference to baseline; CK^Δ%^, creatine kinase expressed as percentage difference to baseline; LDH^Δ%^, lactate dehydrogenase expressed as percentage difference to baseline; C_PBMT_, condition photobiomodulation therapy; G_CWI_, group submitted to cold-water immersion; G_AR_, group submitted to active recovery.

A time effect was found for CK^Δ%^ and IL-10^Δ%^, but not for LDH^Δ%^ and TNFα^Δ%^ blood concentrations. CK^Δ%^ increased at 0.5, 1, and 48-h compared with 72-h, and increased at 0.5-h compared with 24-h [*P* = 0.003; *F*_(8,104)_ = 5.393; *post hoc* *P* ≤ 0.023]. In addition, IL-10^Δ%^ increased over time at 1-h compared with 24 and 48-h [*P* = 0.003; *F*_(8,108)_ = 7.568; *post hoc* *P* = 0.048]. However, there were no interactions (i.e., interaction time^∗^groups) between C_PBMT_, G_AR_, and G_CWI_ for all markers at the same moments [*P* ≥ 0.189; *F*_(8,80)_ ≤ 1.568] (Figure 5). The part-II absolute values of CK, LDH, IL-10, and TNFα blood concentrations are presented in Supplementary Table S2.

Table 3 presents the peak and integral values of CK^Δ%^, LDH^Δ%^, IL-10^Δ%^, and TNFα^Δ%^ reached in C_PBMT_, G_AR_, and G_CWI_. For all blood markers, there were no significant differences between C_PBMT_, G_AR_, and G_CWI_ for peak [*P* ≥ 0.193; *F*_(2,31)_ ≤ 1.734] and integral values [*P* ≥ 0.224; *F*_(2,28)_ ≤ 1.578].

**Table 3:** Comparison between C_PBMT_, G_CWI_, and G_AR_ for peak and integral values of CK, LDH, IL-10, and TNFα.

	C_PBMT_		G_CWI_		G_AR_		ANOVA one-way			
	Peak (Δ%)	Integral (Δ%⋅h)	Peak (Δ%)	Integral (Δ%⋅h)	Peak (Δ%)	Integral (Δ%⋅h)	Peak		Integral	
							*P*-value (F)	Df	*P*-value (F)	Df
CK	139.7 ± 40.7	7902.5 ± 2170.3	162.8 ± 78.5	8316.5 ± 3627.9	157.7 ± 94.3	8560.5 ± 5189.3	0.74 (0.310)	2, 26	0.93 (0.079)	2, 32
LDH	129.1 ± 23.2	7427.3 ± 1332.8	146.9 ± 46.1	7869.8 ± 1536.9	185.0 ± 113.4	7995.2 ± 2470.1	0.19 (1.734)	2, 31	0.78 (0.781)	2, 25
IL-10	184.5 ± 110.7	7956.7 ± 2642.4	219.2 ± 139.1	10112.2 ± 3580.7	185.9 ± 93.8	7672.1 ± 3857.5	0.72 (0.338)	2, 32	0.22 (1.578)	2, 28
TNFα	111.3 ± 23.1	6498.7 ± 983.0	127.9 ± 38.6	7044.5 ± 1623.8	127.3 ± 50.8	7650.0 ± 2019.0	0.56 (0.587)	2, 30	0.32 (1.202)	2, 26


*IL-10, interleukin 10; TNFα, tumor necrosis factor alpha; CK, creatine kinase; LDH, lactate dehydrogenase; C_*PBMT*_, condition photobiomodulation therapy; G_*CWI*_, group submitted to cold-water immersion; G_*AR*_, group submitted to active recovery; Δ%, percentage alteration related to baseline; Δ%⋅h, percentage alteration related to baseline per hour; Df, degrees of freedom.*

**FIGURE 6:**
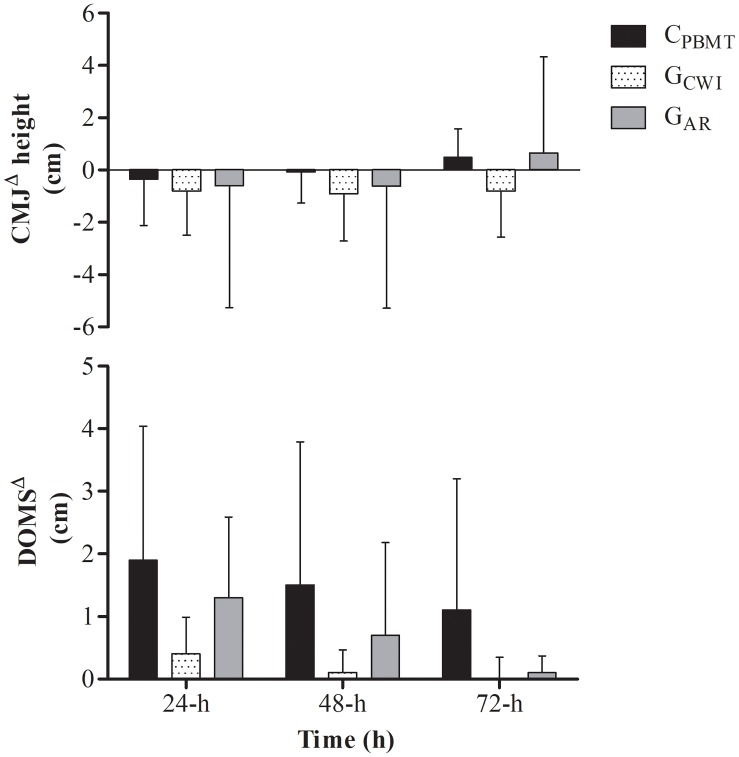
DOMS^Δ^ performance and DOMS^Δ^ for C_PBMT_, G_AR_, and G_CWI_ at 24, 48, and 72-h after double SIT session. DOMS^Δ^, delay onset muscle soreness expressed as difference to baseline; CMJ^Δ^, countermovement jump performance expressed as difference to baseline; C_PBMT_, condition photobiomodulation therapy; G_CWI_, group submitted to cold-water immersion; G_AR_, group submitted to active recovery.

Similar results were found for DOMS^Δ^ and CMJ^Δ^. There was a time effect for DOMS^Δ^ [i.e., DOMS^Δ^ decreased at 48 and 72-h compared with 24-h] [*P* = 0.001; *F*_(4,62)_ = 11.478; *post hoc* *P* ≤ 0.005], but not for CMJ^Δ^ [*P* = 0.253; *F*_(4,62)_ = 1.404], while no interaction was found between groups for either parameter [*P* = 0.314; *F*_(4,62)_ = 1.215 and *P* = 0.264; *F*_(4,62)_ = 1.343, respectively] (Figure 6).

All raw data of inflammation and muscle damage markers, delayed onset muscle soreness, and countermovement jump performance are shown in Supplementary Data Sheet S1.

## Discussion

Some recovery strategies have been used to accelerate muscle recovery in sport routines (Barnett, 2006), however, despite their wide use, several doubts remain in the literature about the effectiveness of these methods. To the best of our knowledge, this is the first study to investigate the effects of PBMT on muscle recovery before SIT sessions using systemic blood markers, muscle performance, and DOMS, and compare it with CWI and AR. The main finding of the current study was the lack of effect of PBMT on muscle recovery compared with C_PLA_, as well as the fact that PBMT did not demonstrate better effects than AR or CWI. Therefore, our initial hypothesis was refuted. The time effects found (Supplementary Figures S3, S4) in the current study only show changes in variable behavior independent of the experimental treatment and therefore do not demonstrate effectivity of any isolated intervention.

Initially, it should be mentioned that there were no differences in workload or metabolic stress induced by the double SIT session in both studies (I and II), indicating that all volunteers presented similar damage induction in all conditions. Consequently, the double SIT elicited increases in CK (≈57%), LDH (≈42%), IL-10 (≈86%), and TNFα (≈24%) blood concentrations in both studies and this increase was not different between conditions.

The process of muscle recovery may be monitored through systemic inflammatory marker kinetics such as cytokines (e.g., interleukins), and is usually accompanied by a decrease in muscle exercise performance and an increase in DOMS (Peake et al., 2017). Interleukins such as IL-10 and TNFα play an important role in the muscle recovery process and their concentration may give an indication of the inflammatory status (Petersen and Pedersen, 2005). In this way, it has been hypothesized that PBMT applied before or after exercise sessions may alter these inflammatory responses due to a decreased effect of reactive oxygen and nitrogen species on cell membranes (Powers and Jackson, 2008), increased activity of satellite cells (Ben-Dov et al., 1999; Shefer et al., 2002), and increased ATP levels (Ferraresi et al., 2015), resulting in better inflammation control and resolution.

Ferraresi et al. (2012) reported evidence of the supposed effect of PBMT on inflammation, which is supported mainly by animal model studies and *in vitro* assay results, focusing predominantly on rehabilitation. Among the few studies that investigated systemic inflammation after exercise with humans, Zagatto et al. (2016) verified only trivial to moderate effect sizes of PBMT on IL-10, IL-1β, and TNFα in young athletes after water polo training sessions, while Aver Vanin et al. (2016) verified a reduction only in IL-6 blood concentration after exercise-induced muscle damage. Therefore, it is clear that there is little evidence to support the beneficial effect of PBMT on inflammation triggered by exercise in humans and our findings indicate that when performed after high-intensity efforts, PBMT as applied in the present investigation has no significant effect on systemic inflammation (see Figure 2).

Decreases in blood CK concentration through PBMT is well reported in the literature (Aver Vanin et al., 2016; De Marchi et al., 2017) while its effect on LDH has been little investigated. Although there is no specific evidence that this mechanism actually occurs, decreases in blood CK and LDH concentrations are often related to the supposed effect of PBMT on hydroxyl radical production in muscle cells, thus reducing the damage caused in the sarcolemma and extravasation of intracellular content to the blood flow (Ferraresi et al., 2012). However, in the present study, PBMT was not able to decrease CK and LDH blood concentrations, and neither were the CWI and AR which presented similar results to PBMT. It should be noted that our results were consistent (i.e., neither damage marker changed) and corroborate with other investigations that also did not verify changes in LDH concentration using PBMT (De Marchi et al., 2012; Zagatto et al., 2016).

The conflicting results in the literature may be explained by the different doses or energy used (Ferraresi et al., 2012). In this way, many authors have made efforts to clarify the dose response effect of PBMT on exercise performance (Dellagrana et al., 2018) and muscle recovery indices (Aver Vanin et al., 2016), however, the optimal dose is still unclear. In the present investigation a dose of 600 J was applied, which is higher than other recent studies (Aver Vanin et al., 2016; Zagatto et al., 2016). However, considering that several lower limb muscles are active during cycling (Hug and Dorel, 2009), and that several application points were required to radiate the entire area, our doses were high mainly due to the application area. Therefore, when the dose per diode irradiation area was relativized the values were ≈ 1.5–4.5 J/cm^2^, close to the dose proposed by Ferraresi et al. (2012) for decreasing muscle damage (≈ 1.0–2.5 J/cm^2^).

Recent studies have observed beneficial effects of PBMT on lower and upper limb isometric maximum voluntary contraction (Aver Vanin et al., 2016; De Marchi et al., 2017) and upper limb one-repetition maximum tests (Felismino et al., 2014) after exercise-induced damage protocols. However, in the present study there were no significant effects of PBMT on CWJ performance compared to C_PLA_, G_CWI_, and G_AR_. These results are in agreement with our inflammatory marker findings, which may produce loss in muscle performance (Peake et al., 2017). In the present study CMJ was used due to its efficacy to detect performance loss after high-intensity exercise sessions (Claudino et al., 2017) and the insignificant effect of the jump, a brief effort, on muscle damage parameters.

In the present study, PBMT was not able to decrease DOMS after the double SIT while CWI and AR were not better than PBMT. Therefore, the results of the present study agree with our findings on inflammation, muscle damage, and performance. However, our results do not corroborate with recent studies that verified beneficial effects using PBMT or when associating PBMT with cryotherapy (de Paiva et al., 2016; De Marchi et al., 2017). It is worth mentioning that the cryotherapy method performed by these authors had no accurate temperature control (ice bag intervention). Machado et al. (2016) in a recent review verified that CWI effectiveness on DOMS is dependent on the temperature (≈ 11–15°C). Therefore, more studies with humans and a well-controlled CWI temperature are necessary to clarify these conflicting results.

The main limitation of the present study is that muscle biopsies were not performed to determine intramuscular recovery parameters. However, in view of the number of blood collections (i.e., 11 in part-I and 6 in part-II) this type of procedure proved unfeasible.

Therefore, our results indicate that PBMT use after acute high-intensity efforts has no effect on muscle recovery. In addition, although the literature suggests the potential changes generated by CWI on muscle recovery mainly due to decreased DOMS (Machado et al., 2016), in the present investigation the CWI was not different to PBMT in any recovery index. Similarly, although AR is a popular recovery method in sports routines (Barnett, 2006), this method also was not different from PBMT, which had no effect on recovery. However, it should be mentioned that some findings of the present study do not agree with previous studies, providing evidence that further studies on this same theme are needed to elucidate the real effects of PBMT.

In summary, PBMT had no effect on inflammation, muscle damage, CMJ performance, or DOMS and was not better than CWI or AR on these recovery indices.

## Author Contributions

EM, FSL, FM, and AMZ conceived and designed the experiments. EM and AMZ performed the experiments. EM, FSL, ASZ, and AMZ analyzed the data. All authors contributed to reagents, materials, and analysis tools and wrote the manuscript.

## Conflict of Interest Statement

The authors declare that the research was conducted in the absence of any commercial or financial relationships that could be construed as a potential conflict of interest.

## Supplementary Material

Figure S1 Performance parameters and lactate kinetics concentration before, during and after the double SIT session. SIT, sprint interval training; BW, bout work; PP, peak power; Wingate test; TW, total work; PP, peak power; MP, mean power; C_PBMT_, photobiomodulation therapy condition; C_PLA_, placebo condition.

Figure S2 Performance parameters and lactate kinetics concentration before, during and after the double SIT session. SIT, sprint interval training; BW, bout work; PP, peak power; Wingate test; TW, total work; PP, peak power; MP, mean power; C_PBMT_, condition photobiomodulation therapy; G_CWI_, group submitted to cold-water immersion; G_AR_, group submitted to active recovery.

Figure S3 Main time effect of blood concentration of IL-10^Δ%^, TNFα^Δ%^, CK^Δ%^, and LDH^Δ%^ (mean ± SD) in part-I and part-II. IL-10^Δ%^, interleukin 10 expressed as percentage difference to baseline; TNFα^Δ%^, tumor necrosis factor alpha expressed as percentage difference to baseline; CK^Δ%^, creatine kinase expressed as percentage difference to baseline; LDH^Δ%^, lactate dehydrogenase expressed as percentage difference to baseline. ^a^*p* < 0.05 compared with 0.5-h. ^c^*p* < 0.05 compared with 24-h. ^d^*p* < 0.05 compared with 48-h. ^e^*p* < 0.05 compared with 72-h.

Figure S4 Main time effect of DOMS^Δ^ performance and CMJ^Δ^ (mean ± SD) in part-I and part-II. DOMS^Δ^, delay onset muscle soreness expressed as difference to baseline; CMJ^Δ^, countermovement jump performance expressed as difference to baseline. ^d^*p* < 0.05 compared with 48-h. ^e^*p* < 0.05 compared with 72-h.

Table S1 Blood concentration of IL-10, TNFα, CK, and LDH (mean ± SD) expressed in absolute values for C_PBMT_ and C_PLA_. IL-10, interleukin 10; TNFα, tumor necrosis factor alpha; CK, creatine kinase; LDH, lactate dehydrogenase; CPBMT, photobiomodulation therapy condition; CPLA, placebo condition; Df, degrees of freedom.

Table S2 Blood concentration of IL-10, TNFα, CK, and LDH (mean ± SD) expressed in absolute values and the effect size of C_PBMT_, G_AR_, and G_CWI_ at baseline, 30 min, 1, 24, 48, and 72 h. L-10, interleukin 10; TNFα, tumor necrosis factor alpha; CK, creatine kinase; LDH, lactate dehydrogenase; CPBMT, photobiomodulation therapy condition; GCWI, group submitted to cold-water immersion intervention; GAR, group submitted to active recovery; Df, degrees of freedom.

Data Sheet S1 Raw data of inflammation and muscle damage markers, delayed onset muscle soreness, and countermovement jump performance.

## Abbreviations

Δ%: percentage of difference to baseline
AR: active recovery
BW: bout work
CK: creatine kinase
CK^Δ%^: creatine kinase concentration percentage of difference to baseline
CMJ: countermovement jump
CMJ^Δ^: countermovement jump height normalized by baseline
C_PBMT_: photobiomodulation therapy condition
C_PLA_: placebo condition
CWI: cold-water immersion
DOMS: delay onset muscle soreness
DOMS^Δ^: delay onset muscle soreness perception normalized by baseline
G_AR_: active recovery group
G_CWI_: cold-water immersion group
GXT: graded exercise test
IL-10: interleukin-10
IL-10^Δ%^: interleukin-10 concentration percentage of difference to baseline
LDH: lactate dehydrogenase
LDH^Δ%^: lactate dehydrogenase concentration percentage of difference to baseline
LED: light emitting diode
MAP: maximal aerobic power
MP: mean power
PBMT: photobiomodulation therapy
PP: peak power
SIT: sprint interval training
TNFα: tumor necrosis factor-α
TNFα^Δ%^: tumor necrosis factor-αconcentration percentage of difference to baseline
TT: total work
VAS: visual analog scale
O_2_: oxygen uptake
O_2peak_: peak oxygen uptake
