# Supplementary figures and images for: Photobiomodulation by Led Does Not Alter Muscle Recovery Indicators and Presents Similar Outcomes to Cold-Water Immersion and Active Recovery
Source: Front Physiol. 2019 Jan 14;9:1948. doi: 10.3389/fphys.2018.01948 (PMC6339932; doi:10.3389/fphys.2018.01948)

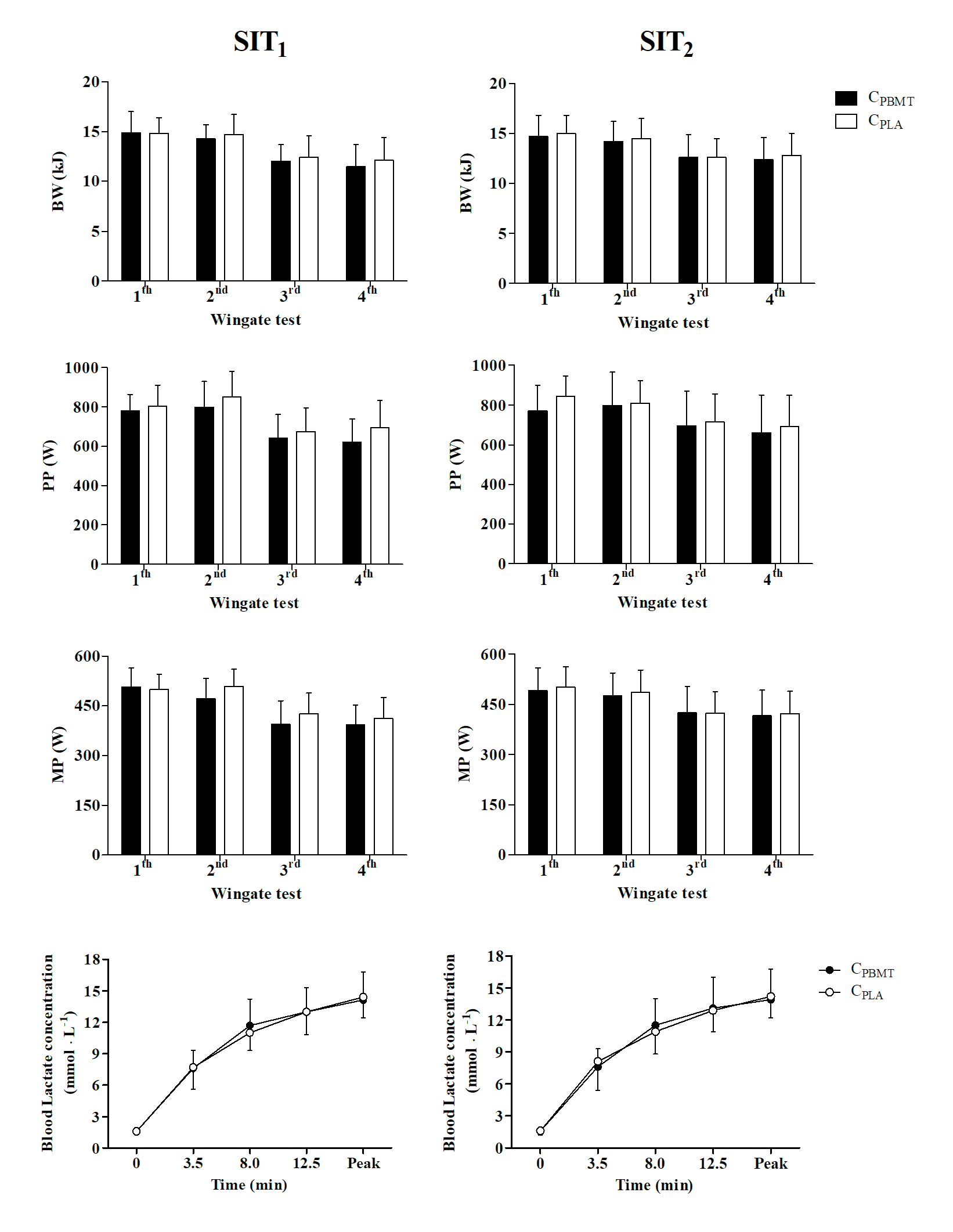

Supplement: Figure S1 — Performance parameters and lactate kinetics concentration before, during and after the double SIT session. SIT, sprint interval training; BW, bout work; PP, peak power; Wingate test; TW, total work; PP, peak power; MP, mean power; CPBMT, photobiomodulation therapy condition; CPLA, placebo condition. [file Image_1.TIF]

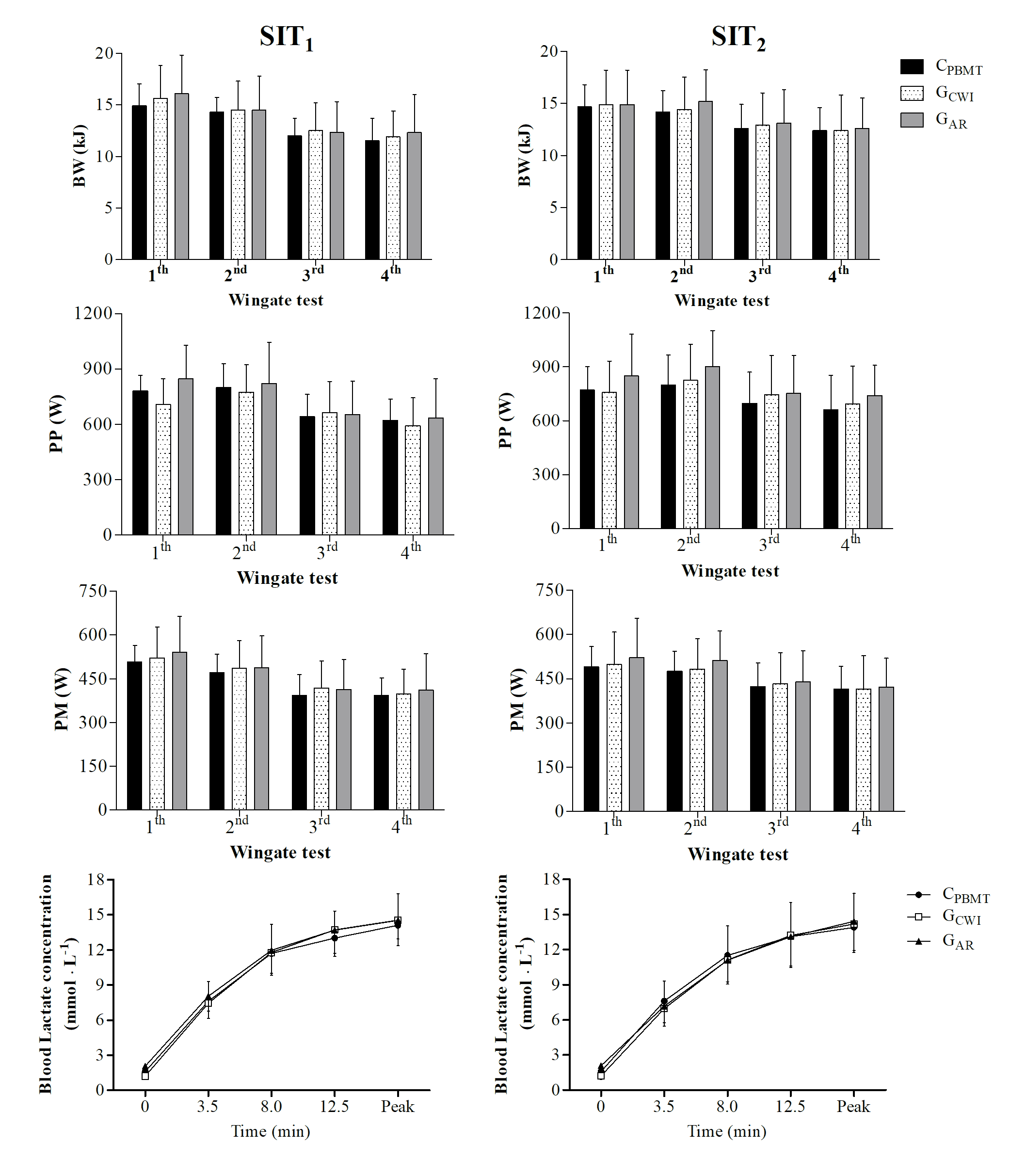

Supplement: Figure S2 — Performance parameters and lactate kinetics concentration before, during and after the double SIT session. SIT, sprint interval training; BW, bout work; PP, peak power; Wingate test; TW, total work; PP, peak power; MP, mean power; CPBMT, condition photobiomodulation therapy; GCWI, group submitted to cold-water immersion; GAR, group submitted to active recovery. [file Image_2.TIF]

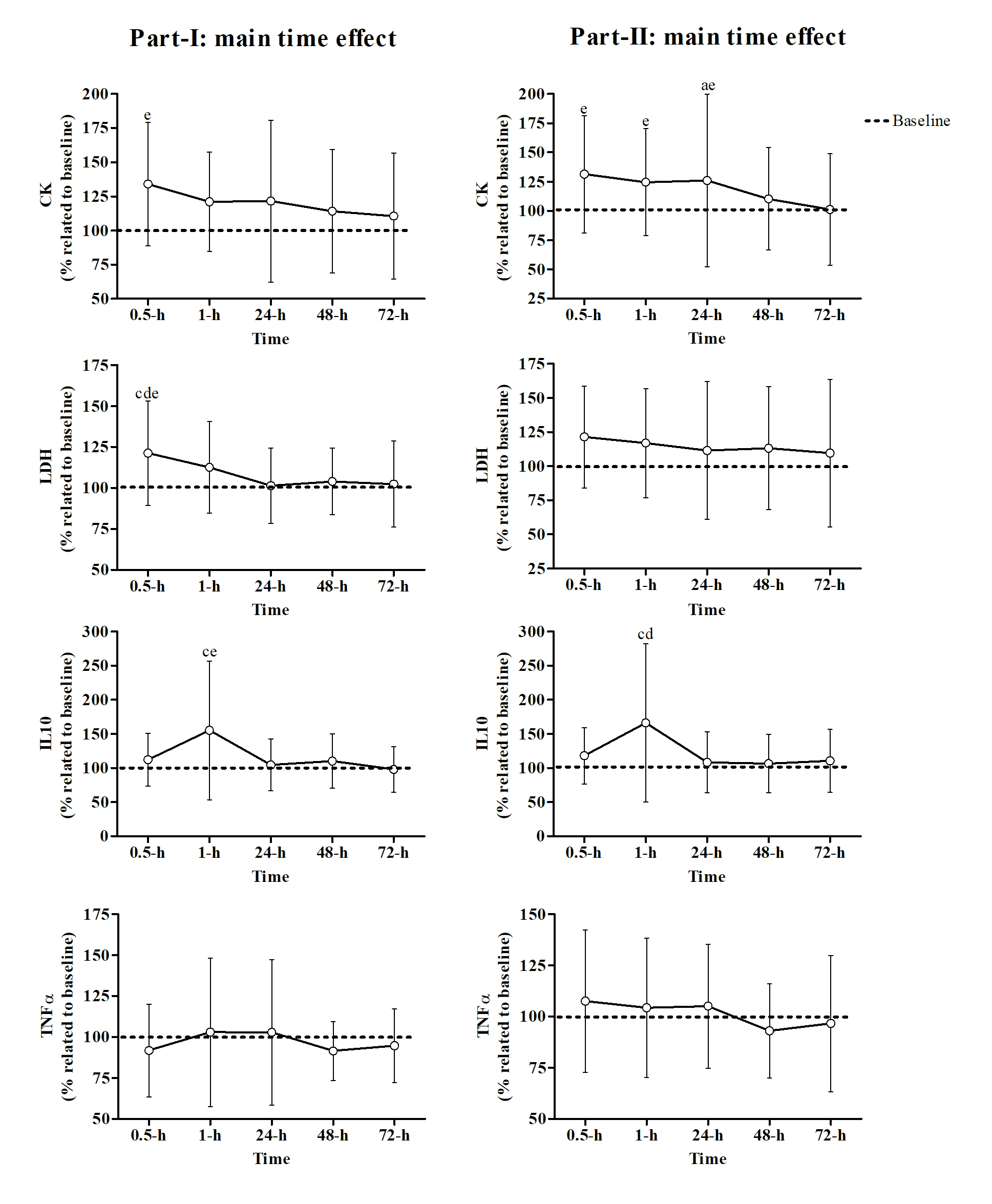

Supplement: Figure S3 — Main time effect of blood concentration of IL-10Δ%, TNFαΔ%, CKΔ%, and LDHΔ% (mean ± SD) in part-I and part-II. IL-10Δ%, interleukin 10 expressed as percentage difference to baseline; TNFαΔ%, tumor necrosis factor alpha expressed as percentage difference to baseline; CKΔ%, creatine kinase expressed as percentage difference to baseline; LDHΔ%, lactate dehydrogenase expressed as percentage difference to baseline. ap < 0.05 compared with 0.5-h. cp < 0.05 compared with 24-h. dp < 0.05 compared with 48-h. ep < 0.05 compared with 72-h. [file Image_3.TIF]

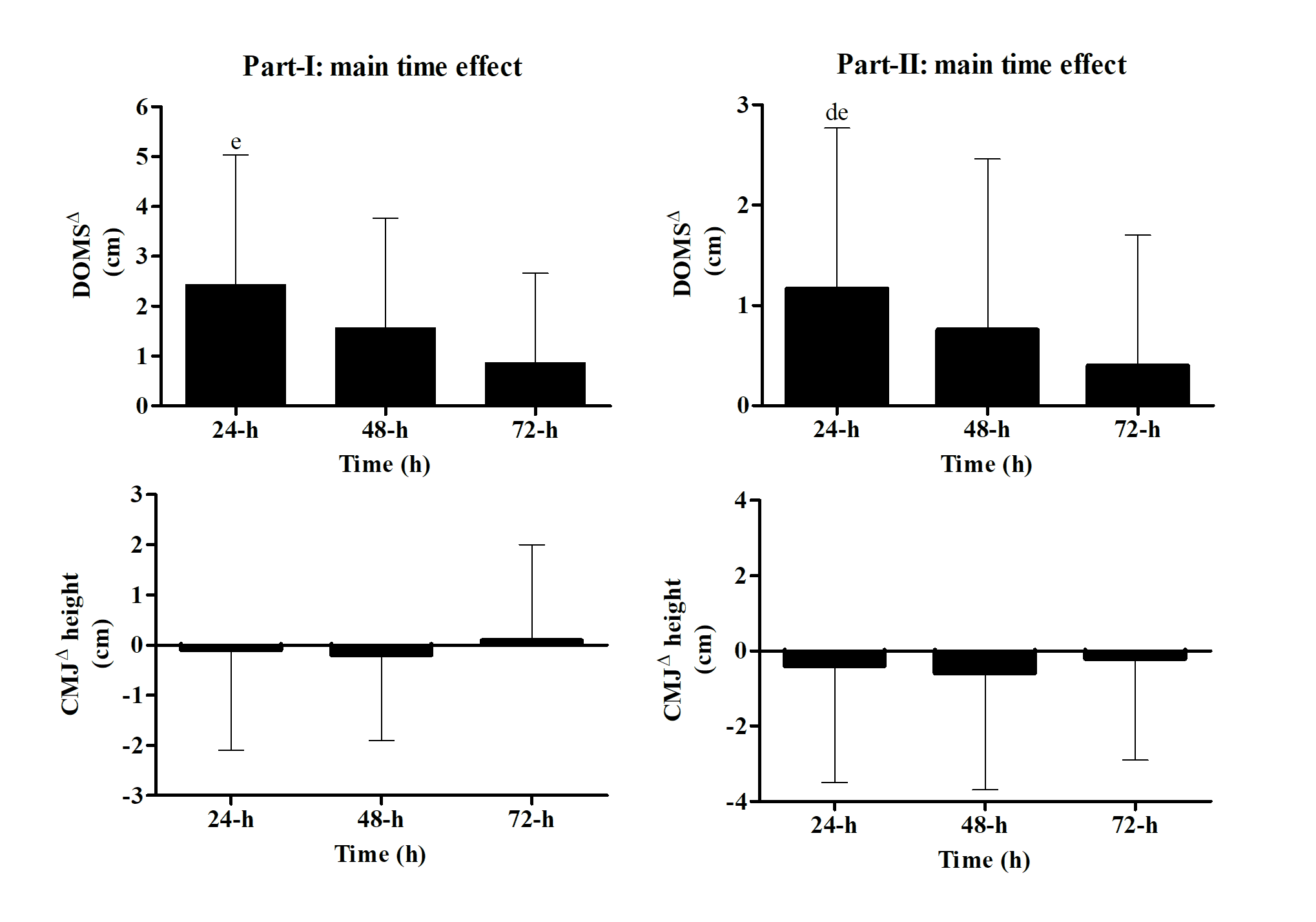

Supplement: Figure S4 — Main time effect of DOMSΔ performance and CMJΔ (mean ± SD) in part-I and part-II. DOMSΔ, delay onset muscle soreness expressed as difference to baseline; CMJΔ, countermovement jump performance expressed as difference to baseline. dp < 0.05 compared with 48-h. ep < 0.05 compared with 72-h. [file Image_4.TIF]
