# Supplementary material for: Photobiomodulation by Led Does Not Alter Muscle Recovery Indicators and Presents Similar Outcomes to Cold-Water Immersion and Active Recovery
Source: Front Physiol. 2019 Jan 14;9:1948. doi: 10.3389/fphys.2018.01948 (PMC6339932; doi:10.3389/fphys.2018.01948)
Supplement: Table S1 — Blood concentration of IL-10, TNFα, CK, and LDH (mean ± SD) expressed in absolute values for CPBMT and CPLA. IL-10, interleukin 10; TNFα, tumor necrosis factor alpha; CK, creatine kinase; LDH, lactate dehydrogenase; CPBMT, photobiomodulation therapy condition; CPLA, placebo condition; Df, degrees of freedom. [file Table_1.DOCX]

Supplementary Table 1. Blood concentration of IL-10, TNFα, CK and LDH (mean ± SD) expressed in absolute values for C_PBMT_ and C_PLA_.

|  | | IL-10 (pg∙mL^-1^) | | TNFα  (pg∙mL^-1^) | CK  (U·L^-1^) | LDH  (U·L^-1^) |
| --- | --- | --- | --- | --- | --- | --- |
| C_PBMT_ | Baseline | | 1.96 ± 1.35 | 84.6 ± 98.3 | 69.6 ± 19.8 | 250.9 ± 54.4 |
|  | 0.5-h | | 2.06 ± 1.16 | 100.0 ± 129.1 | 85.9 ± 25.5 | 302.3 ± 66.8 |
|  | 1-h | | 2.66 ± 1.47 | 90.1 ± 96.6 | 79.3 ± 27.8 | 276.9 ± 90.1 |
|  | 24-h | | 1.73 ± 0.88 | 78.4 ± 58.3 | 73.9 ± 18.5 | 245.0 ± 48.7 |
|  | 48-h | | 1.76 ± 0.91 | 88.3 ± 96.4 | 75.0 ± 23.6 | 262.8 ± 19.4 |
|  | 72-h | | 1.69 ± 0.85 | 96.2 ± 105.9 | 69.3 ± 17.1 | 239.7 ± 45.5 |
| C_PLA_ | Baseline | | 1.96 ± 1.35 | 84.6 ± 98.3 | 69.6 ± 19.8 | 250.9 ± 54.4 |
|  | 0.5-h | | 2.05 ± 1.40 | 90.9 ± 113.9 | 93.0 ± 31.5 | 296.0 ± 50.8 |
|  | 1-h | | 2.34 ± 1.43 | 87.6 ± 107.8 | 83.6 ± 24.3 | 281.8 ± 49.8 |
|  | 24-h | | 2.28 ± 2.49 | 84.0 ± 97.9 | 84.1 ± 28.4 | 246.7 ± 26.0 |
|  | 48-h | | 2.54 ± 2.85 | 81.0 ± 93.6 | 75.0 ± 25.8 | 246.1 ± 33.7 |
|  | 72-h | | 1.85 ± 1.32 | 85.9 ± 99.9 | 75.4 ± 28.5 | 255.2 ± 38.8 |
| ANOVA two-way  (interaction time*groups) | P-value | 0.635 | | 0.259 | 0.529 | 0.635 |
|  | F | 0.490 | | 1.484 | 0.718 | 0.490 |
|  | Df | 5, 50 | | 5, 40 | 5, 50 | 5, 45 |

IL-10, interleukin 10; TNFα, tumor necrosis factor alpha; CK, creatine kinase; LDH, lactate dehydrogenase; C_PBMT_, phototobiomodulation therapy condition; C_PLA_, placebo condition; Df, degrees of freedom.

Supplementary Table 2. Blood concentration of IL-10, TNFα, CK and LDH (mean ± SD) expressed in absolute values and the effect size of C_PBMT_, G_AR_ and G_CWI_ at baseline, 30 min, 1, 24, 48 and 72 h.

|  | | IL-10 (pg∙mL^-1^) | | TNFα  (pg∙mL^-1^) | CK  (U·L^-1^) | LDH  (U·L^-1^) | |
| --- | --- | --- | --- | --- | --- | --- | --- |
| C_PBMT_ | Baseline | | 1.96 ± 1.35 | 84.6 ± 98.3 | 69.6 ± 19.8 | 250.9 ± 54.4 | |
|  | 0.5-h | | 2.06 ± 1.16 | 100.0 ± 129.1 | 85.9 ± 25.5 | 302.3 ± 66.8 | |
|  | 1-h | | 2.66 ± 1.47 | 90.1 ± 96.6 | 79.3 ± 27.8 | 276.9 ± 90.1 | |
|  | 24-h | | 1.73 ± 0.88 | 78.4 ± 58.3 | 73.9 ± 18.5 | 245.0 ± 48.7 | |
|  | 48-h | | 1.76 ± 0.91 | 88.3 ± 96.4 | 75.0 ± 23.6 | 262.8 ± 19.4 | |
|  | 72-h | | 1.69 ± 0.85 | 96.2 ± 105.9 | 69.3 ± 17.1 | 239.7 ± 45.5 | |
| G_CWI_ | Baseline | | 2.47 ± 3.4 | 53.6 ± 66.8 | 161.2 ± 111.7 | | 333.3 ± 56.0 |
|  | 0.5-h | | 2.69 ± 3.3 | 57.9 ± 73.2 | 177.9 ± 76.4 | | 379.5 ± 89.0 |
|  | 1-h | | 2.58 ± 2.2 | 46.2 ± 51.3 | 176.0 ± 79.3 | | 368.3 ± 73.7 |
|  | 24-h | | 2.10 ± 1.8 | 39.0 ± 51.3 | 173.5 ± 77.9 | | 355.5 ± 96.6 |
|  | 48-h | | 2.32 ± 2.0 | 46.8 ± 58.1 | 171.5 ± 80.5 | | 349.8 ± 117.4 |
|  | 72-h | | 2.05 ± 2.0 | 33.2 ± 41.1 | 134.6 ± 62.3 | | 349.5 ± 89.0 |
| G_AR_ | Baseline | | 2.34 ± 3.2 | 77.6 ± 184.2 | 152.4 ± 113.2 | | 296.2 ± 100.9 |
|  | 0.5-h | | 2.72 ± 4.1 | 85.1 ± 197.6 | 162.3 ± 63.6 | | 354.0 ± 100.7 |
|  | 1-h | | 3.29 ± 3.9 | 97.8 ± 224.3 | 163.2 ± 64.3 | | 400.6 ± 202.5 |
|  | 24-h | | 2.34 ± 3.3 | 89.8 ± 205.4 | 148.6 ± 60.7 | | 326.8 ± 101.4 |
|  | 48-h | | 2.26 ± 3.1 | 93.5 ± 208.9 | 127.7 ± 37.3 | | 318.0 ± 79.0 |
|  | 72-h | | 2.99 ± 4.1 | 88.7 ± 229.3 | 127.3 ± 76.2 | | 311.2 ± 96.6 |
| ANOVA two-way  (interaction time*groups) | P-value | 0.879 | | 0.283 | 0.465 | 0.814 | |
|  | F | 0.400 | | 1.294 | 0.910 | 0.463 | |
|  | Df | 10, 140 | | 10, 105 | 10, 140 | 10, 130 | |

IL-10, interleukin 10; TNFα, tumor necrosis factor alpha; CK, creatine kinase; LDH, lactate dehydrogenase; C_PBMT_, phototobiomodulation therapy condition; G_CWI_, group submitted to cold-water immersion intervention; G_AR_, group submitted to active recovery; Df, degrees of freedom.
