# Supplementary material for: Photobiomodulation by Led Does Not Alter Muscle Recovery Indicators and Presents Similar Outcomes to Cold-Water Immersion and Active Recovery
Source: Front Physiol. 2019 Jan 14;9:1948. doi: 10.3389/fphys.2018.01948 (PMC6339932; doi:10.3389/fphys.2018.01948)
Supplement: Data Sheet S1 — Raw data of inflammation and muscle damage markers, delayed onset muscle soreness, and countermovement jump performance. [file Data_Sheet_1.pdf]

Supplementary Data Sheet 1. Raw data of inflammation and muscle damage markers, delayed onset muscle soreness, and countermovement jump performance.

CK

Study I

VOLUNTEER	PBMT						PLACEBO				
	Baseline	30min	1h	24h	48h	72h	30min	1h	24h	48h	72h
A	95	99	91	99	121	83	130	112	94	65	71
D	65	55	47	55	41	46	46	48	45	46	55
J	80	86	120	64	75	78	82	95	68	87	94
E	46	90	45	71	69	58	111	64	59	62	77
F	39	61	77	50	67	77	44	60	135	81	59
I	72	57	56	48	50	48	77	79	65	44	54
Y	75	139	131	105	106	101	141	133	84	73	70
Z	58	78	75	69	70	67	107	106		137	154
W	105	120	104	93	81	80	83	85	76	76	83
B	50	86	55	78	47	46	68	71	74	48	51
C	65	65	73	74	81	69	123	68	133	93	53
K	85	95	78	81	92	79	104	82	92	88	84
**mean**	**69,6**	**85,9**	**79,3**	**73,9**	**75,0**	**69,3**	**93,0**	**83,6**	**84,1**	**75,0**	**75,4**
**SD**	**19,8**	**25,5**	**27,8**	**18,5**	**23,6**	**17,1**	**31,5**	**24,3**	**28,4**	**25,8**	**28,5**

Study II

VOLUNTEER	CWI					
	Baseline	30min	1h	24h	48h	72h
PP	128	120	157	130	129	97
AB	220	174	155	142	257	115
XX	204	218	218	233	239	238
ZZ	244	257	281	265	137	92
DD	61	147	118	151	125	128
OO	394	326	321	281	265	240
JJ	259	221	222	216	173	129
KK	90	118	121	151	162	164
FF	41	51	60	43	74	54
WW	65	129	97	67	42	
QQ	67	196	186	229	284	89
**mean**	**161,2**	**177,9**	**176,0**	**173,5**	**171,5**	**134,6**
**SD**	**111,7**	**76,4**	**79,3**	**77,9**	**80,5**	**62,3**

VOLUNTEER	AR					
	Baseline	30min	1h	24h	48h	72h
S	93	113	101	105	123	111
T	381	272	222	160	103	97
L	384	258	231	174	128	43
O	73	79	68	69	66	67
M	70	116	118	285	82	304
R	139	176	160	123	136	
AA	173	185	228	156	136	120
BB	154	165	233	171	158	124
CC	129	217	206	214	207	202
Q	84	105		110	130	
N	58	93	78	72		69
P	91	169	150	144	136	136
**mean**	**152,4**	**162,3**	**163,2**	**148,6**	**127,7**	**127,3**
**SD**	**113,2**	**63,6**	**64,3**	**60,7**	**37,3**	**76,2**

LDH

Study I

VOLUNTEER	PBMT						PLACEBO				
	Baseline	30min	1h	24h	48h	72h	30min	1h	24h	48h	72h
A		459	262		273	240	255	305		272	233
D	210	263	208	221	247	222	342	232	233	236	239
J	313	268	503	259	271	147	263	311	251	212	288
E	203	325	277	265	243	285	367	322	265	242	229
F	175	207	145	177	249	204	309	298	244	181	202
I	210	344	263	287	269	284	243	297	229	277	342
Y	253	318	259	295	285	308	339	284	282	252	230
Z	268	321	326	268	281	273	365	350	276	297	291
W	363	346	314	297	262	257	283	338	246	249	262
B	228	222		265	248	232		213	260		
C	255	267	219	151	231	191	223	224	187	216,5	246
K	282	288	270	210	295	233	267	208	241	273	245
**mean**	**250,9**	**302,3**	**276,9**	**245,0**	**262,8**	**239,7**	**296,0**	**281,8**	**246,7**	**246,1**	**255,2**
**SD**	**54,4**	**66,8**	**90,1**	**48,7**	**19,4**	**45,5**	**50,8**	**49,8**	**26,0**	**33,7**	**38,8**

Study II

VOLUNTEER	CWI					
	Baseline	30min	1h	24h	48h	72h
PP	338	314	335	278	295	512
AB	368	371	363	265	283	232
XX	367	391	367	378	457	386
ZZ	345	327	373	333	330	320
DD	341	381	312	330	332	246
OO	385	389	413	513	647	395
JJ	318	436	402	458	279	289
KK	208	265	283	252	277	448
FF	326	370	556	505	282	300
WW	264	608	311	262	316	
QQ	406	323	336	337		367
**mean**	**333,3**	**379,5**	**368,3**	**355,5**	**349,8**	**349,5**
**SD**	**56,0**	**89,0**	**73,7**	**96,6**	**117,4**	**89,0**

VOLUNTEER	AR					
	Baseline	30min	1h	24h	48h	72h
S	373	368	308	310	390	356
T	430	607	334	288	262	249
L	244	323	282	242	241	231
O	247	287	246	286	359	260
M	139	357	320	501	437	466
R	282	316	325	280	354	
AA	165	194	750	154	147	127
BB	347	323	853	515	311	354
CC	456	399	314	307	274	326
Q	327	326		363	300	
N	199	302	289	302	362	341
P	345	446	386	374	379	402
**mean**	**296,2**	**354,0**	**400,6**	**326,8**	**318,0**	**311,2**
**SD**	**100,9**	**100,7**	**202,5**	**101,4**	**79,0**	**96,6**

IL-10

Study I

VOLUNTEER	PBMT						PLACEBO				
	Baseline	30min	1h	24h	48h	72h	30min	1h	24h	48h	72h
A	5,06	3,76	4,71	2,66	2,54	2,66	5,43	5,98	9,10	10,76	4,51
D	2,06	2,84	4,26	1,59	3,51	1,96	2,21	3,52	1,67	1,97	2,06
J	0,98	1,20	1,43	0,92	0,55	0,52	2,39	2,01	0,51	0,57	0,38
E	1,00	1,41	4,37	1,22	1,23	1,49	1,32	1,61	1,45	1,00	1,40
F	0,65	0,54	0,37	0,92	1,02	0,59	0,67	2,07	1,17	0,98	0,82
Y	0,66	0,95	2,38	0,72	0,94	1,05	0,75	1,46	0,73	0,84	1,07
Z	1,95	2,75	1,84	2,44	1,82	2,09	1,75	1,74	1,26	2,40	1,68
W	2,20	2,88	2,56	1,47	2,07	1,70	1,80	1,61	1,92	2,15	1,52
B	3,70	3,75	4,06	3,60	2,90	3,40	3,59	3,25	4,34	3,43	4,17
C	1,67	1,02	1,26	2,06	1,36	1,42	0,98	0,88	0,82	1,67	1,06
K	1,62	1,61	2,03	1,48	1,37	1,67	1,61	1,58	2,12	2,16	1,71
**mean**	**1,96**	**2,06**	**2,66**	**1,73**	**1,76**	**1,69**	**2,05**	**2,34**	**2,28**	**2,54**	**1,85**
**SD**	**1,35**	**1,16**	**1,47**	**0,88**	**0,91**	**0,85**	**1,40**	**1,43**	**2,49**	**2,85**	**1,32**

Study II

VOLUNTEER	CWI					
	Baseline	30min	1h	24h	48h	72h
PP	1,10	1,80	4,00	1,21	0,89	1,26
JJ	0,62	0,52	0,50	0,52	0,94	0,62
OO	3,65	2,52	2,90	3,22	3,01	2,81
KK	0,49	1,29	0,74	0,70	0,94	0,88
XX	0,10	0,21	0,61	0,20	0,15	0,16
QQ	0,61	0,86	1,20	1,05	0,61	0,66
AB	1,13	1,22	1,83	1,73	1,77	2,45
WW	3,83	4,81	2,75	3,05	4,00	
ZZ	2,35	2,87	3,97	4,32	3,89	1,58
EE	2,14	3,27	2,93		2,95	2,67
DD	12,64	12,21	8,24	5,73	7,29	7,60
FF	0,93	0,68	1,32	1,37	1,40	1,85
**mean**	**2,47**	**2,69**	**2,58**	**2,10**	**2,32**	**2,05**
**SD**	**3,4**	**3,3**	**2,2**	**1,8**	**2,0**	**2,0**

VOLUNTEER	AR					
	Baseline	30min	1h	24h	48h	72h
Q	0,64	0,48		1,11	1,18	
M	0,89	0,80	0,69	1,58	3,94	1,89
P	1,12	1,12	2,51	0,39	0,63	1,26
R	1,16	1,79	2,75	1,49	1,33	
O	1,22	1,02	1,55	0,72	1,04	1,38
N	4,37	5,11	6,17	5,04	1,68	5,75
L	1,05	0,85	1,69	0,56	0,89	0,74
T	0,72	0,85	1,68	0,13	0,31	0,38
CC	0,92	1,25	1,02	1,17	0,58	0,37
S	1,82	1,47	1,49	1,49	1,56	1,66
AA	2,10	2,67	2,51	2,46	2,33	2,71
BB	12,02	15,21	14,17	11,92	11,61	13,73
**mean**	**2,34**	**2,72**	**3,29**	**2,34**	**2,26**	**2,99**
**SD**	**3,2**	**4,1**	**3,9**	**3,3**	**3,1**	**4,1**

TNFa

Study I

VOLUNTEER	PBMT						PLACEBO				
	Baseline	30min	1h	24h	48h	72h	30min	1h	24h	48h	72h
A	165,0	175,4	160,7	144,5	153,3	173,6	194,7	162,8	185,6	178,3	167,9
D	239,4	252,6	223,0	216,6	227,2	257,6	230,4	236,7	241,6	231,9	240,0
J	15,6	12,1	9,1	9,7	9,0	8,5	10,7	10,2	9,8	8,8	8,3
E	23,5	24,6	23,3	23,9	22,8	21,1	24,8	23,7	25,0	24,5	26,1
Y	3,3	2,1	3,3	3,0	3,9	2,2	2,0	2,9	2,2	2,3	4,6
Z	241,1	371,1	241,4	248,3	238,5	244,3	309,8	290,3	221,8	212,1	241,7
W	14,7	11,9	12,6	11,6	12,5	9,9	9,4	10,6	12,8	12,0	11,3
B	0,3	0,3		0,4			0,1	0,8	0,8	0,3	0,3
C	23,3	22,6	25,6	25,4	21,3	23,6	22,2	23,3	25,0	28,8	25,1
K	119,6	127,0	111,6	101,0	105,9	125,3	105,1	114,6	115,7	110,7	134,2
**mean**	**84,6**	**100,0**	**90,1**	**78,4**	**88,3**	**96,2**	**90,9**	**87,6**	**84,0**	**81,0**	**85,9**
**SD**	**98,3**	**129,1**	**96,6**	**94,0**	**96,4**	**105,9**	**113,9**	**107,8**	**97,9**	**93,6**	**99,9**

Study II

VOLUNTEER	CWI					
	Baseline	30min	1h	24h	48h	72h
PP	35,5		39,4	33,7	34,5	34,4
OO	38,3	24,5	23,9	22,7	12,7	24,6
KK	10,5	21,2	11,0	10,1	11,7	11,4
XX	12,6	12,2	16,3	11,6	10,8	11,8
QQ	8,3	10,4	10,7	11,8	8,8	7,1
AB	4,8	5,8	6,2	8,8	3,7	5,4
WW	180,0	205,5	125,3	158,7	160,5	
ZZ	13,4	13,8	17,0	13,9	14,7	11,4
EE	99,0	112,0	103,1		127,2	84,4
DD	175,0	158,9	142,8	106,4	117,8	129,0
FF	12,7	14,5	12,7	12,7	12,7	12,5
**mean**	**53,6**	**57,9**	**46,2**	**39,0**	**46,8**	**33,2**
**SD**	**66,8**	**73,2**	**51,3**	**51,3**	**58,1**	**41,1**

VOLUNTEER	AR					
	Baseline	30min	1h	24h	48h	72h
Q	48,7	59,6		61,3	61,2	
M	27,9	37,2	33,8	33,4		32,3
P	1,9	0,6			0,8	0,7
R	86,3	112,1	109,5	90,7	93,6	
O	14,1	18,3	15,4	13,1	14,6	17,6
N	1,4	2,5	1,0	2,2		1,3
L	7,5	5,2	5,6	6,6	8,2	8,0
T	4,3	6,6	6,3	5,5	5,6	5,7
CC	2,7	1,6	1,5	1,3	2,0	1,4
S	78,5	72,3	70,1	66,9	66,5	75,9
AA	2,9	2,8	6,8	4,7	2,3	6,4
BB	654,6	702,2	727,9	701,9	680,5	737,9
**mean**	**77,6**	**85,1**	**97,8**	**89,8**	**93,5**	**88,7**
**SD**	**184,2**	**197,6**	**224,3**	**205,4**	**208,9**	**229,3**

CMJ - max heigh (cm)

Study I

VOLUNTEER	PBMT basaline	PBMT 24	PBMT 48	PBMT 72	PLACEBO basaline	PLACEBO 24	PLACEBO 48	PLACEBO 72
A	33,3	36	32,3	32	33,3	32,8	34,3	33,2
D	43,1	40,7	41,8	41,3	43,1	40	40,4	40,4
J	30,8	30,4	30,7	30,3	30,8	30,4	31,5	30,9
E	35,1	35,4	36	35,9	35,1	36,4	36,4	38,9
F	39,7	38,9	37	40,7	39,7	41,3	40,3	41,7
I	25,3	26,6	27,6	28,4	25,3	25,5	26,9	26,4
Y	39,3	38	38,3	37,4	39,3	39,7	39,6	39,4
Z	35,4	33,5	34,1	34,2	35,4	36,6	35	36,2
W	33,2	38,9	38,3	38	33,2	34,1	35	34,8
B	34,4	32,3	32,4	32,4	34,4	34,6	33,4	34,7
C	39,1	39,3	36,7	34,8	39,1	35,4	33,3	35
K	34,2	34,7	33,2	33,5	34,2	31,9	31,8	32,1
**mean**	**35,2**	**35,4**	**34,9**	**34,9**	**35,2**	**34,9**	**34,8**	**35,3**
**SD**	4,68	4,17	3,90	3,95	4,68	4,45	3,98	4,38

Study II

VOLUNTEER	CWI basaline	CWI 24	CWI 48	CWI 72
PP	29,8	29,3	27,2	28,5
AB	35,2	34,2	34,8	34,6
XX	49,3	45,3	46,2	49,3
ZZ	41,3	41,5	41,8	44
DD	33,5	33,5	34,1	33,3
OO	43,9	44,9	41,5	40,8
EE	26,4	25,7	29	28,1
JJ	34,1	33,8	34,6	32
KK	32,9	30,2	30,8	30,7
FF	32	33,8	32,4	
WW	30,8	30,4	28,5	29,7
QQ	29,6	26,5	27,2	27,2
**mean**	**34,9**	**34,1**	**34,0**	**34,4**
**SD**	6,66	6,59	6,23	7,24

VOLUNTEER	AR basaline	AR 24	AR 48	AR 72
S	31,6	28,3	27,8	30,9
T	44,1	40,7	43,3	43,7
L	37,2	34,4	33,4	36,4
O	42,8	43,1	45,6	45,3
M	39,3	37,5	39	39,7
R	33,9	31,6	32,5	31,6
AA	45,6	39,8	36,4	39,7
BB	46,7	53,3	50,5	50,4
CC	35,8	45,6	43	43,6
Q	40,1	38,6	40,4	
N	52,1	47,1	45,9	50,9
P	32,3	34,4	36,3	36,3
**mean**	**40,1**	**39,5**	**39,5**	**40,8**
**SD**	6,33	7,04	6,54	6,75

DOMS (mm)

Study I

VOLUNTEER	PBMT				PLACEBO			
	basal	24	48	72	basal	24	48	72
A	0	7,3	6,5	5,3	0	6,5	7,2	4,2
D	0	1,8	0,4	0,3	0	9,2	3,8	0,6
J	0	0,7	0,3	0	0	0,8	0	0
E	0,2	0,4	0	0,2	0,2	3,3	0,1	0,1
F	0,9	4,5	2,2	0,5	0,9	3,8	2,3	1,1
I	0,6	4,1	6,4	4,5	0,6	6,3	4,5	2,5
Y	0,3	1	0,6	0,1	0,3	0,4	0,5	0,1
Z	1,6	4,3	2,8	1,2	1,6	6,1	3,1	0,6
W	1,8	3,3	4,2	6,1	1,8	4	2,8	3,8
B	0	0,2	0	0	0	0,1	0	0
C	0	0	0	0	0	0	0	0
K	0	0,2	0,1	0,1	0	0,8	0,6	0,1
**mean**	**0,5**	**2,3**	**2,0**	**1,5**	**0,5**	**3,4**	**2,1**	**1,1**
**SD**	**0,65**	**2,34**	**2,49**	**2,33**	**0,65**	**3,08**	**2,30**	**1,54**

Study II

VOLUNTEER	CWI			
	basaline	24	48	72
PP	0,2	0,4	0,1	0
AB		1,6	0,8	0,8
XX	0	0,8	0	0
ZZ	0	0	0	0
DD	0	0	0	0
OO		0	0	0
EE	0,8	0,9	0	0
JJ	0,2	1,7	0,7	0,4
KK	0	0,2	0,1	0
FF	0	0,3	0,1	0
WW	0	0	0	0
QQ	0	0	0,2	0
**mean**	**0,1**	**0,5**	**0,2**	**0,1**
**SD**	0,25	0,62	0,28	0,25

VOLUNTEER	AR			
	basaline	24	48	72
S	0	1	0	0
T	0	0	0	0
L	0	0	0	0
O	0,1	3,6	2,2	0,4
M	0	3	0,6	0
R	0	0	0	0
AA	0,3	0,1	0	0
BB	0	1,8	0	0
CC	0	2,3	0	0
Q	0,1	0,2	0,2	
N	0	2	4,9	
P		1,6	1,1	0,8
**mean**	**0,0**	**1,3**	**0,8**	**0,1**
**SD**	0,09	1,27	1,47	0,27
